# SIRT1 induces epithelial-mesenchymal transition by promoting autophagic degradation of E-cadherin in melanoma cells

**DOI:** 10.1038/s41419-017-0167-4

**Published:** 2018-01-26

**Authors:** Ting Sun, Lin Jiao, Yangxia Wang, Yan Yu, Liang Ming

**Affiliations:** 1 Key Clinical Laboratory of Henan Province, Department of Clinical Laboratory, The First Affiliated Hospital of Zhengzhou University, No.1 Jianshe Road East, Zhengzhou, 450052 China; 2 Department of Laboratory Medicine, West China Hospital, Sichuan University, Chengdu, 610041 China; 3 State Key Laboratory of Oncology in South China, Cancer Center, Sun Yat-sen University, Guangzhou, 510060 China

## Abstract

Melanoma is highly metastatic, and understanding of its molecular mechanism is urgently needed for the development of therapeutic targets and prognostic assessment for metastatic melanoma. SIRT1 is a nicotinamide adenine dinucleotide (NAD^+^)-dependent protein deacetylase, belonging to the mammalian sirtuin family. It has been reported that SIRT1 is associated with metastasis in various cancers. However, the molecular mechanism of SIRT1 in melanoma metastasis remains to be clarified. Here we report that SIRT1 induces the epithelial–mesenchymal transition (EMT) by accelerating E-cadherin degradation via autophagy and facilitates melanoma metastasis. Initially, we found that SIRT1 expression was frequently elevated in metastatic melanoma compared with primary melanoma. In addition, SIRT1 induced the EMT and promoted cell migration and invasion by decreasing E-cadherin expression. Further work demonstrated that SIRT1 accelerated the autophagic degradation of E-cadherin through deacetylation of Beclin 1. In addition, inhibition of autophagy recovered E-cadherin expression and suppressed cell migration and invasion by delaying the degradation of E-cadherin in SIRT1-overexpressing cells. Overall, our findings reveal a novel molecular mechanism for SIRT1 in melanoma metastasis, indicating that SIRT1 may serve as a viable therapeutic target for metastatic melanoma.

## Introduction

Melanoma is a lethal skin cancer that is highly metastatic^1,2^. Tumor metastasis poses a major barrier to the effective treatment of melanoma. In the United States, the 5-year relative survival rate is 18% for melanoma patients diagnosed at a distant stage^3^. Currently, there is no efficacious treatment for metastatic melanoma. Consequently, understanding of the molecular mechanism that regulates melanoma metastasis is needed for the development of additional therapeutic targets and new drugs for treating this disease.

Tumor metastasis is facilitated by the epithelial–mesenchymal transition (EMT). The EMT is a dynamic and reversible phenotypic switching process that enables polarized epithelial cells to gain characteristics of mesenchymal cell phenotypes as well as enhanced migratory and invasive capacities^4–7^. The EMT converts benign tumors into invasive, metastatic tumors and plays an important role in promoting tumor progression and metastasis^8^. Loss of expression of E-cadherin, a cell–cell adhesion molecule that is mainly expressed in epithelial cells, is the foundation for the activation of the EMT^9,10^. There are many transcription factors that work as E-cadherin repressors. Snail, ZEB, E47, and KLF8 factors bind to and directly repress the activity of the E-cadherin promoter, whereas factors such as Twist and FoxC2 repress E-cadherin transcription indirectly^11^. In addition, expression of mesenchymal markers N-cadherin and vimentin are increased in the EMT.

SIRT1 belongs to the class III histone deacetylase family, depending on nicotinamide adenine dinucleotide (NAD^+^) for its deacetylase activity^12,13^. Studies have shown that SIRT1 is involved in many physiological processes, including cellular metabolism, senescence and stress responses^14–16^. Over the past few decades, studies have increasingly shown that SIRT1 is involved in the initiation and progression of various cancers^17,18^. SIRT1 potentially plays multiple roles in altering cellular processes, such as cell proliferation, apoptosis, invasion and metastasis^19–21^. Previous studies have revealed that SIRT1 promotes the EMT and metastasis in chondrosarcoma, osteosarcoma, oral squamous cell carcinoma, hepatocellular carcinoma, pancreatic cancer and colorectal cancer^22–27^. SIRT1 is also essential for lamellipodium extension and the migration of melanoma cells^28^. However, the precise regulatory mechanisms and signaling pathways underlying the SIRT1-mediated EMT and melanoma metastasis remain unclear.

Autophagy is an evolutionarily conserved lysosome-dependent cellular catabolic degradation pathway and is vital in the maintenance of cellular homeostasis^29,30^. It has been proved that autophagy plays an important role in cancer onset and metastasis^31^. Studies have shown that autophagy plays a dual role in regulating the EMT. However, the exactly molecular mechanism of autophagy involved in EMT and melanoma metastasis is still unclear and need to be further studied.

In our study, we hypothesized that SIRT1 promotes melanoma metastasis by inducing the EMT. We found a correlation between the level of SIRT1 expression and tumor metastasis in melanoma tissues and studied the potential mechanism responsible for the SIRT1-mediated metastatic effect. Our findings suggest that SIRT1 can induce the EMT by promoting the autophagy-linked lysosomal degradation of E-cadherin, the master suppressor of the EMT. Therefore, our study demonstrates a novel mechanism for SIRT1 in promoting EMT in melanoma cells and provides a potential therapeutic target for metastatic melanoma.

## Results

### SIRT1 expression is frequently elevated in metastatic melanoma

**Fig. 1:**
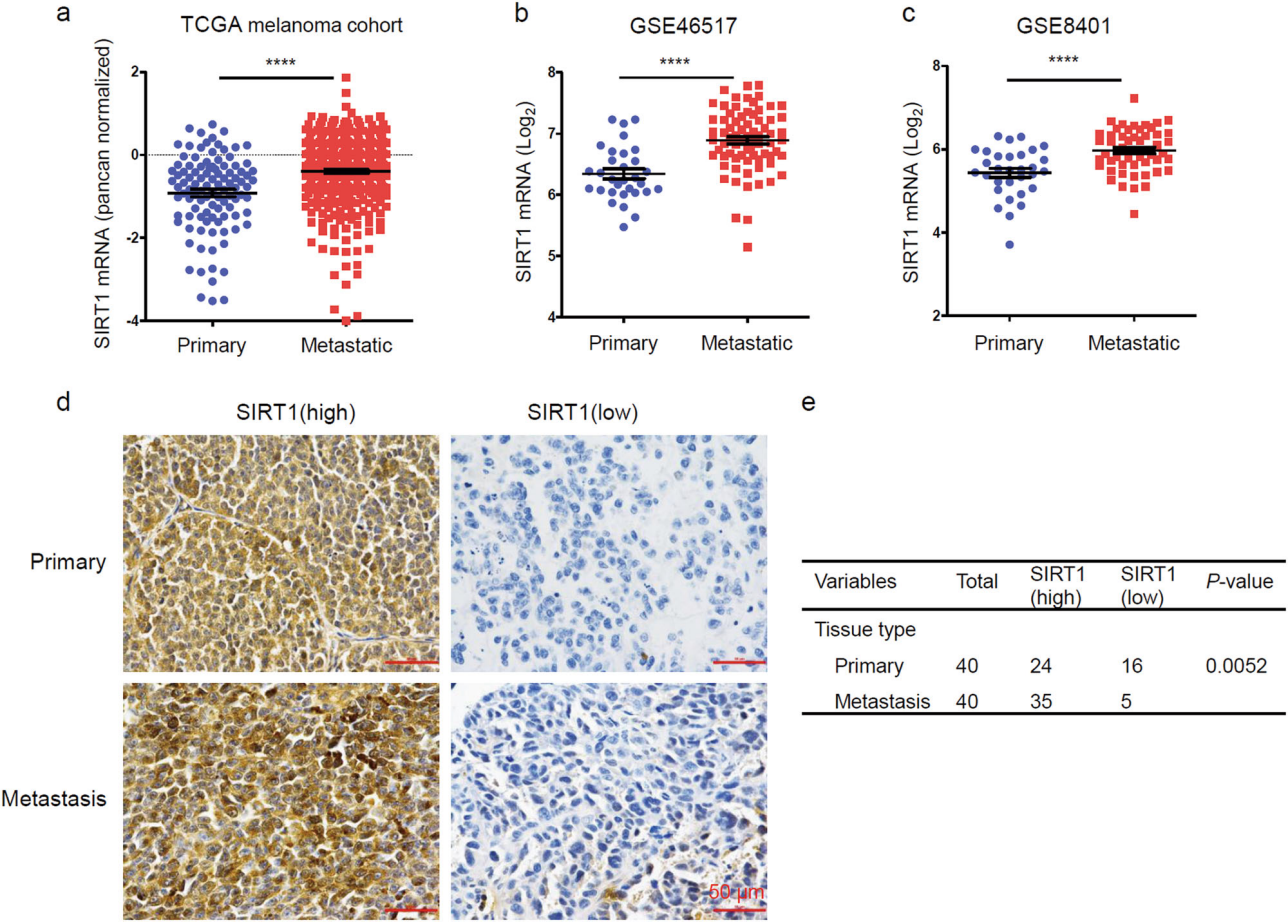
Overexpression of SIRT1 in metastatic melanoma **a** A analysis of SIRT1 mRNA expression in primary melanoma (*n* = 103) and metastatic melanoma (*n* = 370). The RNA data were downloaded from the TCGA database. **b**, **c** Analysis of SIRT1 mRNA levels in primary and metastatic melanoma tissues from the GSE46517 and GSE8401 public data sets. **d** The expression of SIRT1 in the primary and metastatic melanoma tissues was analyzed by IHC. **e** The relationship between SIRT1 expression and the melanoma tissue type was evaluated using Pearson’s *χ*^2^ test

Previous research has shown that SIRT1 expression is upregulated in human melanoma cells and tissues^32^. To validate that SIRT1 is further upregulated in metastatic melanoma, we analyzed melanoma RNA-sequencing data from the TCGA database and found that the SIRT1 mRNA level was higher in metastatic melanoma (*n* = 370) than in primary melanoma controls (*n* = 103) (Fig. 1a). We also analyzed two published microarray data sets of SIRT1 in GEO (nos. 46517 and 8401) and found consistent results, showing that SIRT1 expression was further increased in metastatic melanoma compared with primary melanoma (Fig. 1b, c). To validate the protein expression of SIRT1 in metastatic melanoma, a tissue microarray containing 40 melanoma metastasis samples and 40 primary malignant melanoma samples was stained, and analyzed for SIRT1 protein expression. A higher level of SIRT1 protein was found in 87.5% (35/40) of melanoma metastases, whereas 60% (24/40) of primary melanoma samples exhibited an elevated protein level (Fig. 1d, e). There was a significant enhancement of SIRT1 staining in melanoma metastases, as calculated using Pearson’s *χ*^2^ statistical analysis. These results suggest that SIRT1 expression is elevated in metastatic melanoma.

### SIRT1 promotes the EMT and metastatic potential in melanoma cells

**Fig. 2:**
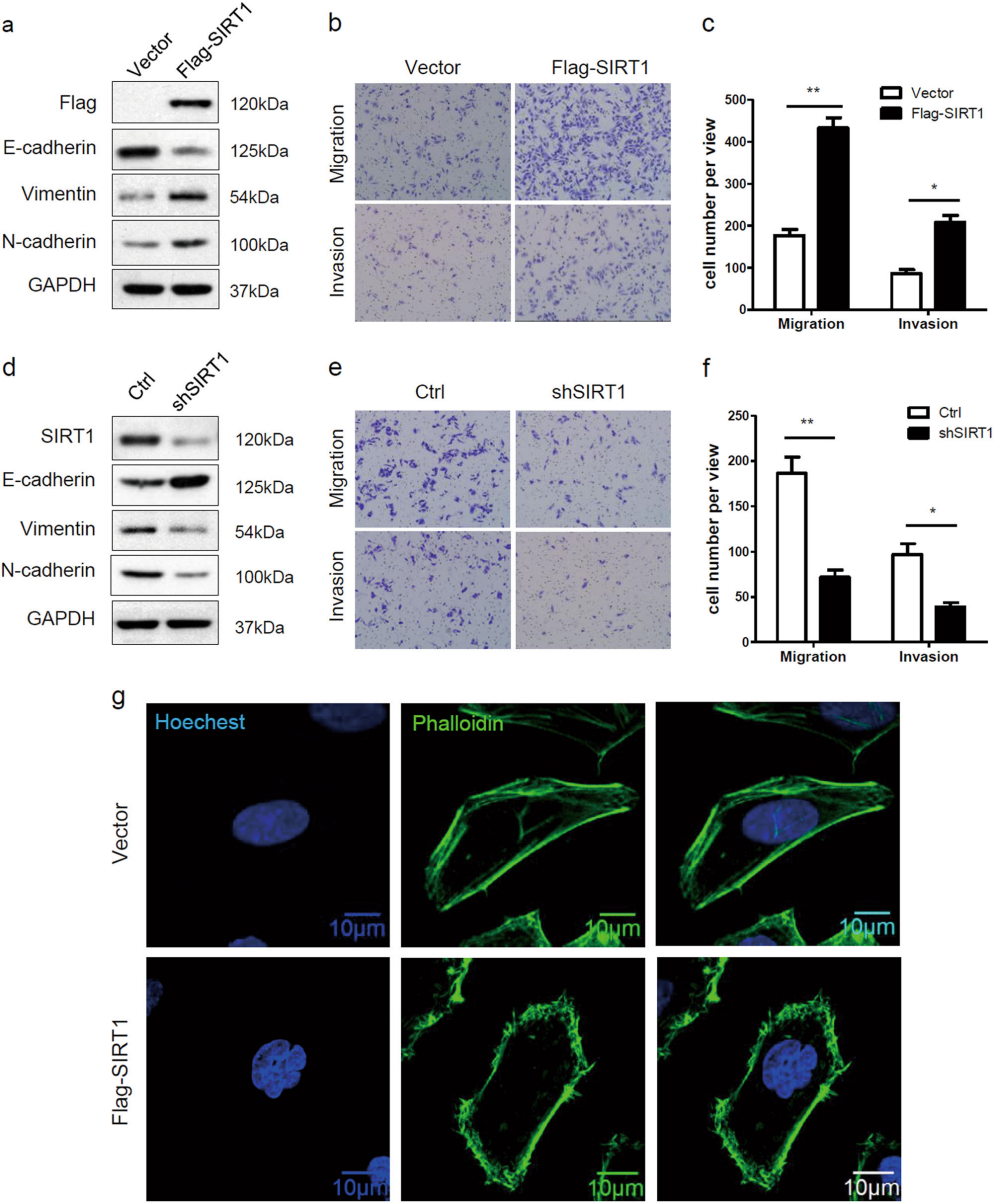
SIRT1 promotes the EMT and increase metastatic potential in melanoma cells **a** Sk-Mel-28 cells stably expressing vector or Flag-SIRT1 were harvested and protein expression was detected by western blot analysis. **b**, **c** Sk-Mel-28 cells stably expressing vector or Flag-SIRT1 were plated in the upper chamber of the transwell filters for 24 h. Cells migrating to the underside of the transwell insert were then counted. **d** A375-shSIRT1 and control cells were harvested, and protein expression was detected by western blot analysis. **e**, **f** A375-shSIRT1 and control cells were plated in the upper chamber of the transwell filters for 24 h. Cells migrating to the underside of the transwell insert were then counted. **g** Immunofluorescence staining of actin filaments (Phalloidin) in Sk-Mel-28 cells stably expressing either vector or Flag-SIRT1. Bar = 10 μm

We further investigated whether SIRT1 promotes the metastatic potential of melanoma cells by regulating the EMT. We established Sk-Mel-28 cells stably expressing either vector or Flag-SIRT1 and examined changes in EMT markers. SIRT1 overexpression reduced the expression of the epithelial marker E-cadherin and enhanced the expression of the mesenchymal markers vimentin and N-cadherin (Fig. 2a). We also verified the cells’ migration and invasion capacities in vitro. Compared with the vector control, SIRT1 overexpression promoted melanoma cell migration and invasion, as indicated by a transwell migratory assay and Matrigel invasion assay, respectively (Fig. 2b, c). To further confirm whether high endogenous SIRT1 expression contributes to migration and invasion, SIRT1 was stably knocked down in A375 cells via a lentivirus-based approach. The results showed that SIRT1 knockdown decreased mesenchymal marker expression, whereas E-cadherin was upregulated slightly (Fig. 2d). Moreover, SIRT1 knockdown significantly weakened melanoma cell migration and invasion compared with the control (Fig. 2e, f). Previous studies have shown that cell migration is strongly supported by reorganization of actin cytoskeleton^28^. We also examined the effect of SIRT1 on the architecture of cytoskeleton by staining the actin filaments. We found that SIRT1 overexpression increased lamellipodium extension in Sk-Mel-28 cells (Fig. 2g). It suggests that SIRT overexpression promotes cytoskeleton rearrangement, which is a necessary event for EMT and cell migration. These data indicate that SIRT1 enhances the EMT in melanoma cells and promotes migration and invasion in vitro.

### SIRT1 accelerates the degradation of E-cadherin through the lysosomal pathway

**Fig. 3:**
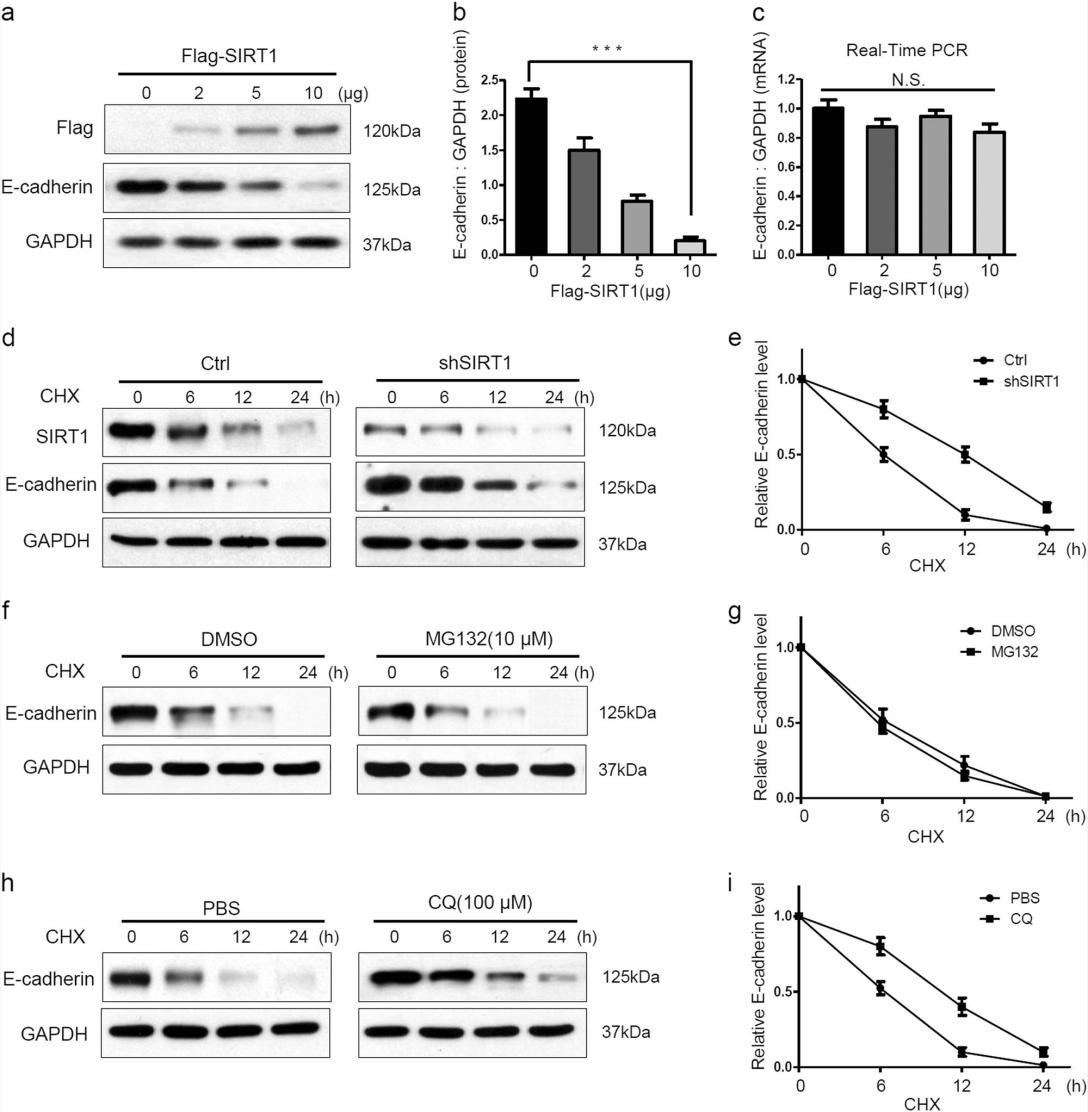
SIRT1 promotes the lysosomal degradation of E-cadherin **a**–**c** A375 cells were transfected with the indicated concentrations of Flag-SIRT1 for 24 h. Protein expression were analyzed by western blotting and E-cadherin mRNA expression was analyzed by RT-PCR. **d**, **e** A375-shSIRT1 and control cells were treated with CHX (20 μM) for the indicated times, and the protein levels were detected by western blotting. **f**, **g** A375 cells were treated with MG132 (10 μM) for 2 h, and then also treated with CHX (20 μM) for the indicated times. The protein levels were detected by western blotting. **h**, **i** A375 cells were treated with CQ (100 μM) for 12 h, and then CHX (20 μM) was added for the indicated times. The protein level were detected by western blotting. The bars represent mean ± SE (*n* = 3). N.S nonsignificant; CHX cycloheximide; CQ chloroquine

Because the loss of E-cadherin expression is the foundation of the EMT, we speculated that SIRT1 stimulates the EMT by downregulating E-cadherin. We next investigated the exact mechanism of SIRT1-mediated E-cadherin reduction in melanoma cells. It was found that SIRT1 overexpression decreased the expression of E-cadherin protein but not E-cadherin mRNA in a concentration-dependent manner (Fig. 3a–c). Therefore, we hypothesized that SIRT1 might decrease E-cadherin protein expression by accelerating its degradation. In the present of cycloheximide (CHX), a protein synthesis inhibitor, SIRT1 knockdown slowed E-cadherin degradation in A375 cells (Fig. 3d, e). The proteasome-based proteolytic pathway and the lysosomal pathway are the major pathways for protein degradation in cells. We next examined which pathway participates in SIRT1-induced E-cadherin degradation. Inhibition of the proteasome by MG132 did not affect the degradation of E-cadherin (Fig. 3f, g). However, inhibition of lysosome function by chloroquine (CQ) delayed the degradation of E-cadherin in A375 cells (Fig. 3h, i). Therefore, these findings suggest that SIRT1 accelerates the degradation of E-cadherin through the lysosomal pathway.

### SIRT1 stimulates autophagic degradation of E-cadherin through deacetylation of Beclin 1

**Fig. 4:**
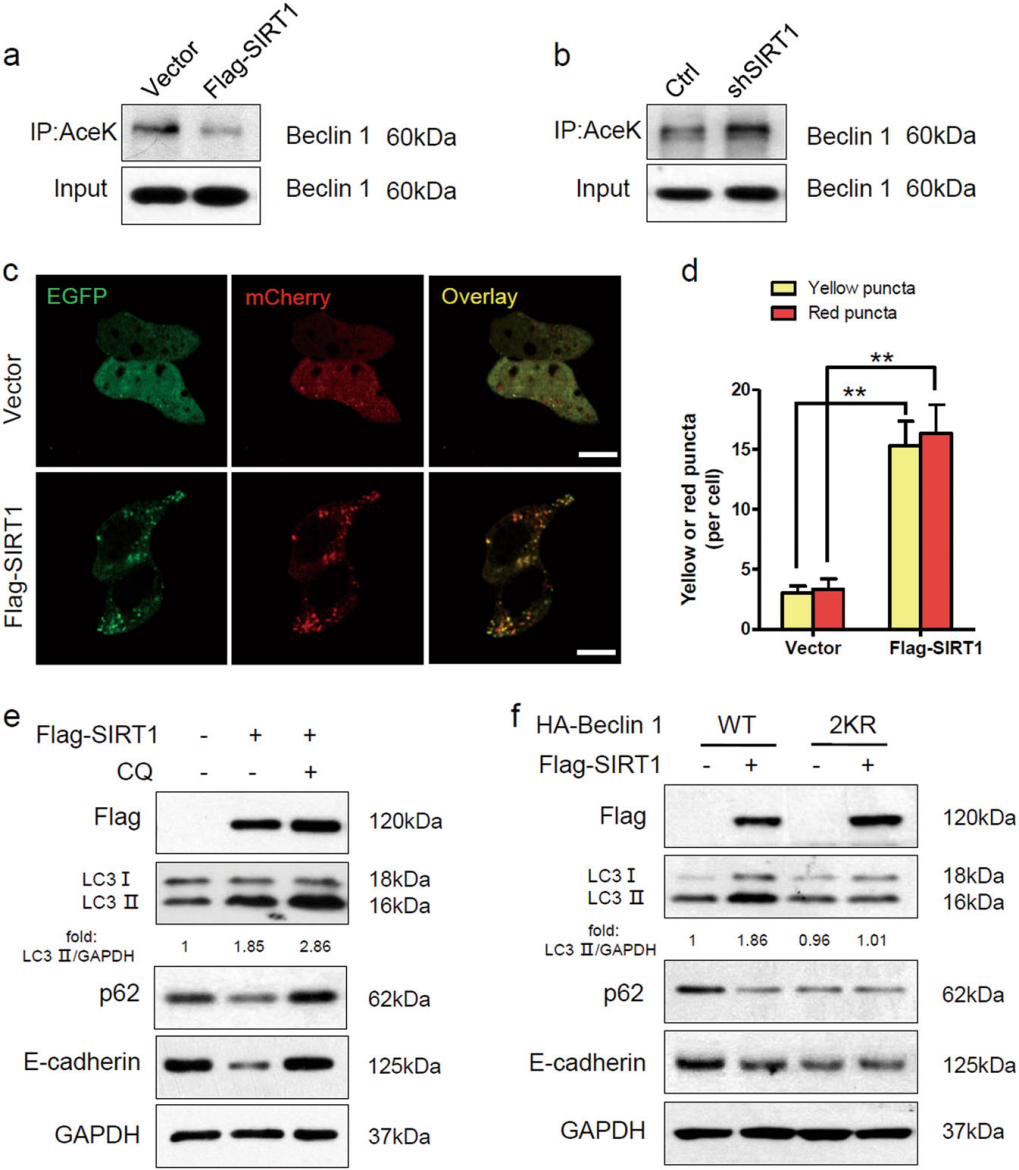
SIRT1 deacetylates Beclin 1 and stimulates autophagic degradation of E-cadherin **a** The protein expression in Sk-Mel-28 cells stably expressing vector or Flag-SIRT1 were extracted and immunoprecipitated using an anti-acetylated lysine antibody. **b** The protein expression in A375-shSIRT1 and control cells were extracted and immunoprecipitated using an anti-acetylated lysine antibody. **c** Sk-Mel-28 cells stably expressing vector or Flag-SIRT1 were transfected with mCherry-EGFP-LC3 for 24 h. The cells were then imaged by confocal microscopy. Scale bars, 5 μm. **d** Quantitation of the red and yellow puncta in c. The bars represent mean ± SE of 50 cells; three independent experiments, **P < 0.01 (Student’s *t* test). **e** Sk-Mel-28 cells expressing either vector or Flag-SIRT1 were treated with or without CQ (100 mM) for 12 h, and protein expression was measured by western blotting. **f** Sk-Mel-28 cells expressing either vector or Flag-SIRT1 were transfected with HA-tagged Beclin 1 (WT, 2KR) for 24 h, and protein expression was then measured by western blotting. AceK, acetylated lysine

Proteins are degraded by the lysosome through either the autophagic or the endocytic pathway. Our previous studies have shown that SIRT1 can deacetylate Beclin 1 at lysine 430 and lysine 437, and that Beclin 1 deacetylation enhances autophagic degradation^33^. We speculated that autophagy might participate in the lysosomal degradation of E-cadherin, and we investigated the role of SIRT1 in regulating autophagy in melanoma cells. We first examined the effect of SIRT1 on Beclin 1 acetylation in melanoma cells. We found that SIRT1 overexpression significantly decreased Beclin 1 acetylation, whereas SIRT1 knockdown increased acetylation of Beclin 1 (Fig. 4a, b). Furthermore, we employed EGFP-mCherry-LC3 plasmid to monitor the autophagic flux regulated by SIRT1^34^. In cells transfected with vector, only weak signals of EGFP and mCherry were observed in the cytoplasm. However, SIRT1 overexpression induced a large number of yellow and red puncta, suggesting more autophagosome and autolysosome formation (Fig. 4c, d). To further evaluate the effect of SIRT1 on autophagic degradation, we detected the expression of LC3, p62 and E-cadherin by western blot analysis. The conversion of the soluble form of LC3 (LC3I) to the lipidated form (LC3II) is a sign of autophagy activation, and p62 is recognized as a mark of autophagic degradation^35^. Sk-Mel-28 cells stably expressing vector or Flag-SIRT1 were treated with CQ or not. We found that SIRT1 overexpression enhanced the expression of LC3II and decreased the level of p62 in Sk-Mel-28 cells. However, the autophagy inhibitor CQ blocked the SIRT1-induced degradation of p62 (Fig. 4e). The changes in the protein level of E-cadherin were consistent with the changes in p62 expression. We then transfected wild-type or 2KR-mutant (lysine 430 and lysine 437 were mutated to arginine, which cannot be acetylated) Beclin 1 into Sk-Mel-28 cells. The 2KR mutation decreased the level of p62 because Beclin 1 deacetylation promoted autophagic degradation. SIRT1 overexpression promoted the degradation of p62 in cells with wild-type Beclin 1. However, in cells with the Beclin 1 2KR mutation, p62 degradation was not enhanced by SIRT1 overexpression. Correspondingly, the changes in the protein level of E-cadherin were consistent with the changes in p62 expression. Taken together, our data indicate that SIRT1 promotes E-cadherin degradation by autophagy through deacetylation of Beclin 1 in melanoma cells.

### SIRT1 induces the EMT and increases metastatic potential by autophagic degradation of E-cadherin

**Fig. 5:**
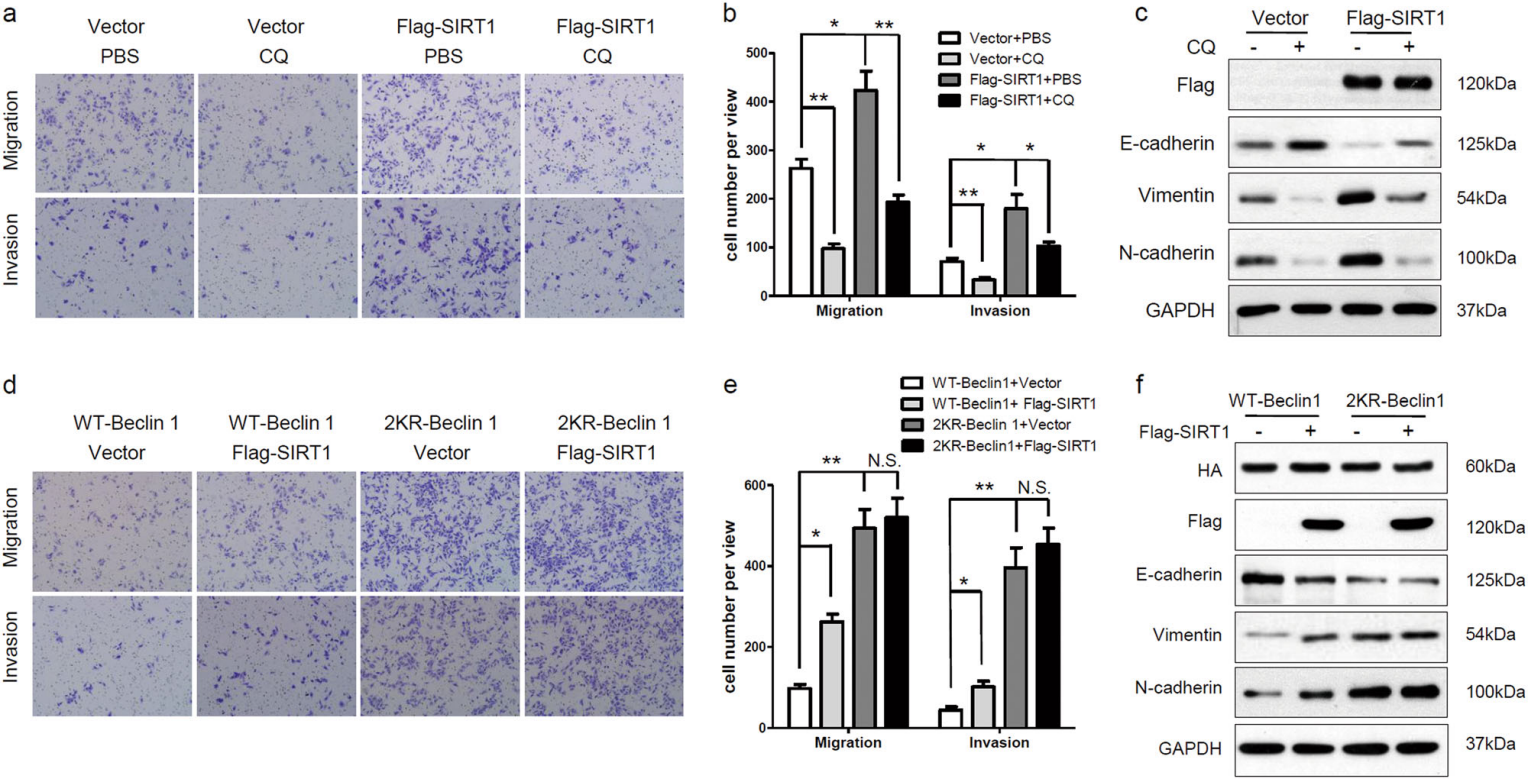
SIRT1 promotes cell migration and invasion by autophagic degradation of E-cadherin **a**, **b** Sk-Mel-28 cells stably expressing vector or Flag-SIRT1 were plated in the upper chamber of transwell filters for 24 h and then treated with PBS or CQ (100 mM, 12 h). Cells migrating to the underside of the transwell insert were subsequently counted. **c** Sk-Mel-28 cells stably expressing vector or Flag-SIRT1 were treated with PBS or CQ (100 mM, 12 h), and protein expression was measured by western blotting. **d**, **e** Sk-Mel-28 cells stably expressing vector or Flag-SIRT1 were transfected with HA-tagged Beclin 1 (WT, 2KR) for 24 h. Cells were plated in the upper chamber of transwell filters for 24 h, and then cells migrating to the underside of the transwell insert were counted. **f** Sk-Mel-28 cells stably expressing vector or Flag-SIRT1 were transfected with HA-tagged Beclin 1 (WT, 2KR) for 24 h, and protein expression was then measured by western blotting

We next investigated whether SIRT1 induces the EMT through autophagic degradation of E-cadherin. Overexpression of SIRT1 promoted melanoma cell migration and invasion (Figs. 2b and 5a), whereas CQ interrupted the acceleration of migration and invasion of SIRT1-overexpressing cells (Fig. 5a, b). Overexpression of SIRT1 reduced E-cadherin expression and enhanced the expression of vimentin and N-cadherin (Figs. 2a and 5c), whereas CQ increased the expression of E-cadherin and decreased the expression of vimentin and N-cadherin in SIRT1-overexpressing cells (Fig. 5c). Moreover, the Beclin 1 2KR mutation promoted melanoma cell migration and invasion compared with wild-type Beclin 1. SIRT1 enhanced migration and invasion of cells transfected with wild-type Beclin 1, but not cells with 2KR-mutant Beclin 1 (Fig. 5d, e). Correspondingly, the Beclin 1 2KR mutation reduced E-cadherin expression and increased the expression of vimentin and N-cadherin, whereas SIRT1 neither increased E-cadherin expression nor reduced vimentin and N-cadherin expression in 2KR mutant Beclin 1-overexpressing cells (Fig. 5f). These data suggest that SIRT1 induces the EMT and promotes cell migration and invasion by stimulating autophagic degradation.

## Discussion

Tumor metastasis is the leading cause of death in cancer patients. Although unremitting efforts on studying the molecular mechanisms of tumor metastasis, the underlying determinants are still not completely clear. An increasing number of studies have shown that SIRT1 plays a crucial role in tumor metastasis and invasiveness in various cancers. For example, SIRT1 promotes the EMT and metastasis by upregulating Fra-1 expression in colorectal cancer^25^. In the present study, we found that SIRT1 promoted the EMT through deacetylation of Beclin 1 and autophagic degradation of E-cadherin in melanoma cells. Although many researchers have shown that SIRT1 promotes metastasis in various cancers, certain studies have still shown that SIRT1 may serve as a suppressor gene for tumor metastasis. Chen et al. found that SIRT1 inhibited cell migration and invasion in oral squamous cell carcinoma by deacetylating Smad4^24^. In addition, another study found that SIRT1 downregulation promoted metastasis by inducing the EMT in breast cancer^36^. This conflict might result from the diversity of cancer types, signaling pathways, and experimental conditions studied.

Activation of the EMT has been proposed to be essential for tumor invasion and metastasis^5,37^, and reduction of E-cadherin expression is the foundation of the EMT^38^. We found that SIRT1 promotes the degradation of E-cadherin by stimulating autophagic degradation and that inhibition of autophagy by CQ recovered the expression of E-cadherin in SIRT1-overexpressing cells. The interplay between autophagy and EMT in cancer is quite controversial^39^. The induction of autophagy leads to loss of the metastatic phenotype by promoting autophagy-mediated degradation of Snail and Twist, 2 major EMT inducers, in breast cancer cells^8^. Moreover, the induction of autophagy suppresses the EMT and then impairs the migration and invasion of glioblastoma cells^40^. It has been recently reported that cadherin-6 promotes EMT and cancer metastasis by restraining autophagy^41^. Therefore, autophagy would appear to be a negative regulator of the EMT process. However, autophagy can also support EMT by promoting survival of cells. Li and colleagues showed that autophagy can promote hepatocellular carcinoma invasion by activating the EMT^42^. Additionally, a recent study showed that sphingosine kinase 1 (SPHK1) induces the EMT by promoting autophagic degradation of E-cadherin and that attenuation of autophagy impairs the EMT and reduces the migration and invasion of HepG2 cells^43^. Hence, we speculate that the function of autophagy in regulating the EMT is dependent on tumor and tissue type.

The role of SIRT1 in promoting tumor metastasis provides a rational explanation for the poor prognosis of cancer patients with high SIRT1 expression. In this study, we found that SIRT1 expression was high in metastatic melanoma compared with primary melanoma. Our findings support a previous study that demonstrated that SIRT1 is involved in melanoma cell migration and that SIRT1 inhibition prevents melanoma metastasis^28^.

**Fig. 6:**
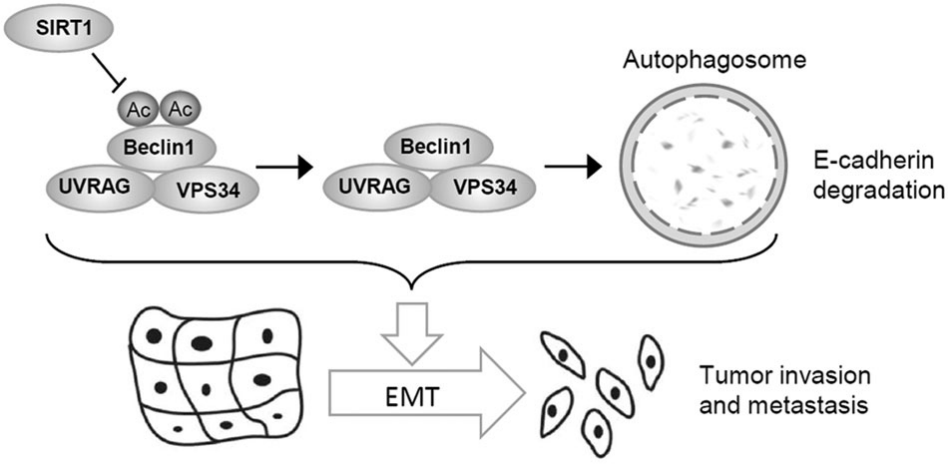
Schematic diagram of the mechanism of SIRT1-mediated autophagic degradation of E-cadherin which stimulates the EMT in melanoma cells

In conclusion, the present study provides conclusive evidence that SIRT1 promotes the EMT by deacetylating Beclin 1 and then accelerates the autophagic degradation of the epithelial marker E-cadherin in melanoma cells (Fig. 6). Our findings define a novel molecular mechanism by which SIRT1 regulates the EMT and metastatic potential of melanoma cells. Therefore, SIRT1 may be a potentially valuable therapeutic target for metastatic melanoma.

## Materials and methods

### Cell culture

The melanoma cell lines Sk-Mel-28 and A375 were purchased from Cell Lines Bank, Chinese Academy of Science (Shanghai, China). The Sk-Mel-28 cells were cultured in complete Dulbecco’s modified Eagle medium (DMEM), and the A375 cells were cultured in high glucose DMEM, supplemented with 10% fetal bovine serum (FBS; Gibco, Grand Island, NY, USA). The cells were cultured at 37 °C in a humid atmosphere with 5% CO_2_.

### Antibodies and reagents

CHX (01810), MG132 (C2211), CQ (C6628) and anti-Flag antibody (F3165) were obtained from Sigma (St. Louis, MO, USA). Anti-E-cadherin antibody (610181) was purchased from BD Biosciences (San Jose, CA, USA). Anti-vimentin (2870), N-cadherin (5741), HA (3724), Beclin 1 (3738), acetylated lysine (9441) antibodies were purchased from Cell Signaling Technology (Danvers, MA, USA). Anti-p62/SQSTM1 (sc-28359) and GAPDH (sc-32233) antibodies were purchased from Santa Cruz Biotechnology (Santa Cruz, CA, USA). Anti-LC3 (NB100–2220) antibody was obtained from NOVUS (Littleton, CO, USA).

### Plasmids and transfection

Flag-SIRT1 was constructed into the pCMV-C-Flag vector (D2632, Beyotime, Shanghai, China) by subcloning. Lipofectamine 2000 was used for plasmid transient transfection according to the manufacturer’s instructions (Invitrogen, Carlsbad, CA, USA). To generate cells stably expressing SIRT1, the vector and the Flag-SIRT1 pCMV plasmid were transfected into Sk-Mel-28 cells for 24 h. Stable transfectants were selected using G418, and a single clone was amplified using a dilution-cloning technique. For stable knockdown, HEK293T cells were transfected with the pLKO or pLKO-shSIRT1 plasmid together with the packaging vectors psPAX2 and pMD2G using Lipofectamine 2000 for 24 h. Culture supernatants were collected, filtered, and diluted with fresh medium (1:1) containing 8 μg/ml polybrene and were then incubated with target cells for another 48 h. Target cells were selected using puromycin (1 μg/ml) for 3 days and were validated by western blotting. The SIRT1 shRNA targeting sequence was as follows: 5′-GGGAATCCAAAGGATAATT-3′.

### Western blotting and immunoprecipitation

As described previously^33^, cells were washed 3 times with PBS and then lysed by 1 × Cell Lysis Buffer (Cell Signaling Technology) containing 1 mM PMSF immediately before use. For acetylation immunoprecipitation of acetylated proteins, cells were lysed using E1A lysis buffer containing 2 mM TSA and 10 mM NAM. Total cell lysates were subjected to immunoprecipitation with the indicated antibodies overnight at 4 °C and then incubated with protein A/G agarose for 12 h at 4 °C. After being washed 3 times, the immunocomplexes were mixed with 2 × SDS loading buffer and boiled for 10 min at 100 °C. The proteins were separated by SDS-PAGE and then transferred to PVDF membranes, followed by blocking in 5% nonfat milk and incubation with primary antibody at 4 °C. After washing with PBST and incubating with secondary antibody, the bands were visualized using ECL.

### Quantitative real-time PCR analysis

Total RNA was extracted from cells using TRIzol reagent, checked for purity and then quantified based on optical density at 260 nm and 280 nm. Reverse transcription was performed using a PrimeScript™ RT reagent kit (Takara). The cDNA was amplified using SYBR Green Master Mix (Roche) on an ABI 7500 Real-Time PCR System. The primer sequences were as follows: E-cadherin Forward: 5′-TGAAGGTGACAGAGCCTCTGG-3′, E-cadherin Reverse: 5′-TGGGTGAATTCGGGCTTGTT-3′, GAPDH Forward: 5′-TGCCAAATATGATGACATCAAGAA-3′, GAPDH Reverse: 5′-GGAGTGGGTGTCGCTGTTG-3′.

### Cell migration and invasion assays

Cell migration was performed in a 24-well plate using transwells (8 μm pore size, Corning Costar, New York, NY, USA). For cell invasion assay, Matrigel (BD Bioscience, San Jose, CA, USA)-coated transwells were used. Cells suspended in 100 μl serum-free medium were added to the upper chamber, and 300 μl medium containing 20% FBS was added to the lower chamber. The plate was incubated at 37 °C in a humidified atmosphere containing 5% CO_2_ for 24 h. The cells that passed through the filters were stained with crystal violet solution and observed under an inverted microscope.

### Tissue microarray slides and immunohistochemistry

In vivo SIRT1 expression was detected by immunohistochemistry (IHC) using tissue microarrays (ME804a, AlenaBio, Xi’an, China), that contained 80 melanoma tissue specimens. The tissues were incubated with primary anti-SIRT1 antibody (1:100, ab32441, Abcam) and biotin conjugated secondary antibody. Hematoxylin was used as the counterstain. Finally, the staining results were evaluated independently by two pathologists. The assessment was classified into 4 grades: negative staining, -; weak staining, +; moderate staining, ++; and strong staining, +++. We defined ++ and +++ as high expression and the others as low expression.

### Statistical analyses

The data are expressed as the mean ± SE. The difference between two groups was assayed using the Student’s *t* test. The correlation between SIRT1 expression and tissue types was evaluated using the Pearson’s *χ*^2^ test. *P < 0.05 and **P < 0.01 were considered statistically significant. Statistical analyses were performed using SPSS and graphs were performed by GraphPad Prism software.
